# Genome‐Wide Silencer Screening Reveals Key Silencer Modulating Reprogramming Efficiency in Mouse Induced Pluripotent Stem Cells

**DOI:** 10.1002/advs.202408839

**Published:** 2025-03-20

**Authors:** Xiusheng Zhu, Lei Huang, Guoli Li, Biao Deng, Xiaoxiao Wang, Hu Yang, Yuanyuan Zhang, Qiuhan Wen, Chao Wang, Jingshu Zhang, Yunxiang Zhao, Kui Li, Yuwen Liu

**Affiliations:** ^1^ Shenzhen Branch Guangdong Laboratory of Lingnan Modern Agriculture Key Laboratory of Livestock and Poultry Multi‐omics of MARA Agricultural Genomics Institute at Shenzhen Chinese Academy of Agricultural Sciences Shenzhen 518124 China; ^2^ State Key Laboratory of Genetic Resources and Evolution Yunnan Laboratory of Molecular Biology of Domestic Animals Kunming Institute of Zoology Chinese Academy of Sciences Kunming 650201 China; ^3^ Kunming College of Life Science University of Chinese Academy of Sciences Kunming 650204 China; ^4^ Guangxi Key Laboratory of Animal Breeding Disease Control and Prevention College of Animal Science and Technology Guangxi University Nanning 530004 China

**Keywords:** *cis*‐regulatory elements, high‐throughput, iPSCs, silencer, Ss‐STARR‐seq

## Abstract

The majority of the mouse genome is composed of non‐coding regions, which harbor numerous regulatory sequences essential for gene regulation. While extensive research focuses on enhancers that activate gene expression, the role of silencers that repress gene expression remains less explored. In this study, the first genome‐wide identification of silencers in the mouse genome is conducted. In mouse embryonic fibroblasts (MEFs) and embryonic stem cells (mESCs), 89 596 and 115 165 silencers are identified, respectively. These silencers are ubiquitously distributed across the genome and are predominantly associated with low‐expression genes. Additionally, these silencers are mainly cell‐specific and function by binding to repressive transcription factors (TFs). Further, these silencers are notably enriched with the histone modification H3K9me3. It is observed that the transformation between dual‐function silencers and enhancers is correlated with intracellular transcription factor concentrations, accompanied by changes in epigenetic modifications. In terms of biological effects, we have identified silencers that can enhance the induction efficiency of MEFs and influence the pluripotency of mESCs. Collectively, this work offers the first comprehensive silencer landscape in the mouse genome and provides strong evidence for the role of silencers in the induction of induced pluripotent stem cells (iPSCs).

## Graphical abstract

This study utilized Ss‐STARR‐seq to construct a genome‐wide silencer map of mouse cells and elucidated the fundamental characteristics of these silencers. These silencers exist in a cell‐specific manner and exhibit unique epigenetic modifications. Through functional experiments, we also discovered that these silencers can regulate gene expression and cellular phenotypes.

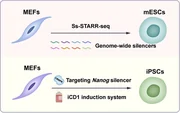

## 1 Introduction

Transcriptional regulation plays a crucial role in organismal growth, development, and cell differentiation. The *cis*‐regulatory information governing gene expression is encoded in the genome in the form of *cis*‐regulatory elements (CREs).^[^ ^1^ ^]^ CREs primarily encompass promoters, enhancers, insulators, and silencers.^[^ ^2^ ^]^ Among these CREs, silencers have been less studied, mainly owing to the lack of distinct defining features.^[^ ^3^ ^]^

Silencers inhibit gene transcription by binding to transcription factors (TFs) known as repressors.^[^ ^4^ ^]^ The first identified silencer, the HMRE element in *Saccharomyces cerevisiae*, functions similarly to enhancers in that its activity is not constrained by distance or orientation.^[^ ^5^ ^]^ The first silencer identified in mammals was in the intronic region of the *CD4* gene, where it exerts a repressive effect on *CD4* gene transcription.^[^ ^6^ ^]^ Subsequently, numerous silencers specific to various tissues and cell types were discovered, and some of their characteristics described.^[^ ^7^ ^]^ Although these studies highlight the critical role of silencers, the systematic identification and characterization of these elements have lagged behind other regulatory components. Therefore, there is an urgent need for high‐throughput methods to identify and analyze the biological characteristics of silencers.

Several high‐throughput experimental methods for the systematic identification of silencers have been reported in recent years. For example, histone H3K27me3 modification is typically associated with transcriptional repression.^[^ ^8^ ^]^ Consequently, Melissa Labs utilized ChIP‐seq for H3K27me3 to identify silencers.^[^ ^9^ ^]^ Their research revealed that regions rich in H3K27me3 exhibit significantly stronger silencing activity. Knockout experiments further confirmed the functional impact of these regions on cellular phenotypes. Similarly, Ngan et al. utilized ChIA‐PET data targeting PRC2 to identify silencers in mouse embryonic stem cells.^[^ ^10^ ^]^ In comparison to approaches measuring epigenetic marks associated with silencers to infer their activity, massive parallel reporter assays provide high‐throughput methods to directly measure silencer activity. For instance, the ReSE screening system uses formaldehyde‐assisted isolation to extract accessible chromatin regions from K562 cells as candidate silencer regions. These are identified by their ability to repress transcription of inducible caspase 9, a protease enzyme that triggers apoptosis upon expression. Following this, high‐throughput sequencing of the surviving cells is conducted to pinpoint the silencer sequences.^[^ ^11^ ^]^ In addition, the modified ATAC‐STARR‐seq method, using a plasmid system, has been reported to successfully identify silencers in B lymphocytes.^[^ ^12^ ^]^ These methods have made significant contributions to the systematic study of silencers. However, due to the vast size of the genome and the screening limitations, current approaches focus on selected regions rather than on performing comprehensive, genome‐wide silencer screenings.

In this study, we applied Ss‐STARR‐seq, a whole‐genome silencer screening method recently developed in our laboratory,^[^ ^13^ ^]^ to mESCs and MEFs, identifying 115 165 and 89 596 silencers, respectively. Following this, we carried out detailed characterization and functional validation of these silencers. These findings are expected to significantly enhance our understanding of silencer mechanisms in mice.

## 2 Results

### 2.1 Identification of Silencers in the Mouse Genome

**Figure 1:**
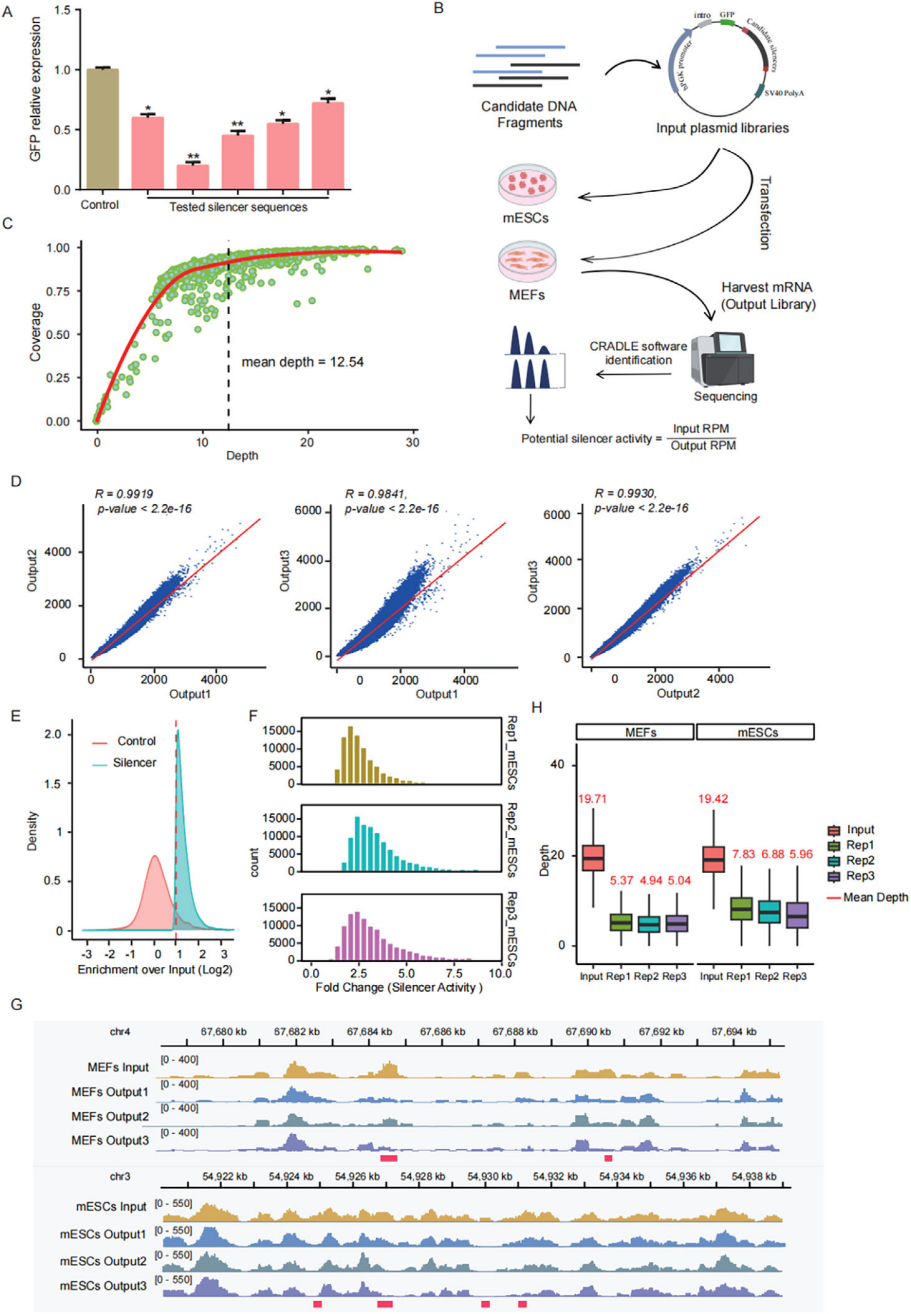
Identification of genome‐wide silencers in mouse cells. A) Validation of five previously reported mouse silencers using Ss‐STARR‐seq, with GFP expression changes on the vector assessed by qPCR, using an empty vector without the added sequences as a control. (*: *p* < 0.05 and **: *p* < 0.01; data are presented as mean values ± SEM; *p* values were obtained using two‐sided Student's *t*‐test, *n* = 3 biologically independent samples). B) The identification process of silencers in mice involves randomly fragmenting genomic DNA into ≈300–400 bp segments, which are then ligated to the Ss‐STARR‐seq vector to construct the input library. This library is subsequently transfected into MEFs and mESCs to generate the output library, followed by high‐throughput sequencing. Finally, silencer regions are identified using the CRADLE software. C) Coverage and sequencing depth of the input library. D) The correlation between the biological repeats of the mESCs output biological library is analyzed. The *x*, *y* coordinate axes represent the number of reads of the genomic bin in different libraries, and the color level indicates the degree of enrichment of the points; output 1/2/3 represents three output libraries with different repeats. E) Ss‐STARR‐seq enrichment at MACS2 peaks and randomly selected GC% and length matched regions. A twofold or higher Ss‐STARR‐seq enrichment over input (red dashed line) is observed in less than 3% of the randomly selected regions and used as a cutoff for significant silencer signal (mESCs). Regions with the same quantity, length, and GC content of peaks are randomly selected in the genome. Fold‐change is calculated and plotted using ggplot2 with R (V4.2.2). F) Distribution of silencer activity (fold change) identified in the three replicate output libraries in mESCs. G) IGV shows the peak signals of mESCs and MEFs silencers in different biological repeated libraries, and red indicates the silencers interval. H) Average sequencing depth of input and output for silencer regions in mESCs and MEFs.

In our previous reports, the PGK promoter was demonstrated to be effective for silencer screening in human cells.^[^ ^13^ ^]^ To evaluate whether the PGK promoter could also be utilized for silencer screening in mouse cells, we cloned previously reported mouse silencers^[^ ^14^ ^]^ into the Ss‐STARR‐seq vector. The results showed that these mouse silencers exhibited significant silencer activity within the Ss‐STARR‐seq vector (**Figure** **1**A), confirming the applicability of the PGK promoter for silencer screening in mouse cells. Subsequently, we utilized Ss‐STARR‐seq for high‐throughput identification of silencers in MEFs and mESCs (Figure 1B). Following the STARR‐seq library construction protocol (see “Experimental Section”), we initially constructed an input library using 300–400 bp fragments(Figure S1A, Supporting Information) of mouse genomic DNA and performed high‐throughput sequencing. The results indicated that the inserted fragments in the input library covered 86.8% of the mouse genome, with an average depth of 12.54× (Figure 1C). This level of complexity is consistent with previous reports that assay CREs on a genome‐wide scale.^[^ ^15^ ^]^ Subsequently, we transfected mESCs and MEFs with the input library plasmids to construct the output libraries (see “Experimental Section”). For both mESCs and MEFs, we generated at least three biological replicates of output libraries. The results showed a high correlation coefficient between the output library replicates from the same cell line (Figure 1D; Figure S1B, Supporting Information), demonstrating the reproducibility of this technique. In addition, the average genome coverage of the output libraries in mESCs and MEFs was 96.4% and 86.7%, with average depths of 12.7× and 8.35×, respectively (Figure S1C, Supporting Information). We analyzed the input and output libraries using CRADLE software,^[^ ^16^ ^]^ specifically designed for identifying silencers in the STARR‐seq system, to identify silencers in MEFs and mESCs. The same procedure was used for 1 million GC%‐matched and size‐matched regions, of which less than 3% exceeded a twofold enrichment (Figure 1E; Figure S1D, Supporting Information). Therefore, we defined silencers with a change greater than twofold as the final set of silencers, identifying 115 165 silencers in mESCs and 89 596 silencers in MEFs. We analyzed the distribution of silencer activities and found that they ranged from 2 to 6, with ≈80% of the activities falling between 2 and 3 (Figure 1F; Figure S1E, Supporting Information). To visualize these silencer regions, we employed the integrated genomics viewer (IGV). The results indicated that the output signal in silencer regions was significantly lower than that in the input, in contrast to non‐silencer regions (Figure 1G). Meanwhile, we also examined the sequence depth of the input and output libraries in the silencer regions. Results showed that in both MEFs and mESCs, the input sequence depth in silencer regions exceeded the average genome‐wide depth. In addition, the sequence depth of the silencer regions in the output libraries was significantly lower than in the input, further supporting the reliability of the identified silencer regions (Figure S1F, Supporting Information).

### 2.2 Genomics and Epigenomics Characteristics of Mouse Silencers

**Figure 2:**
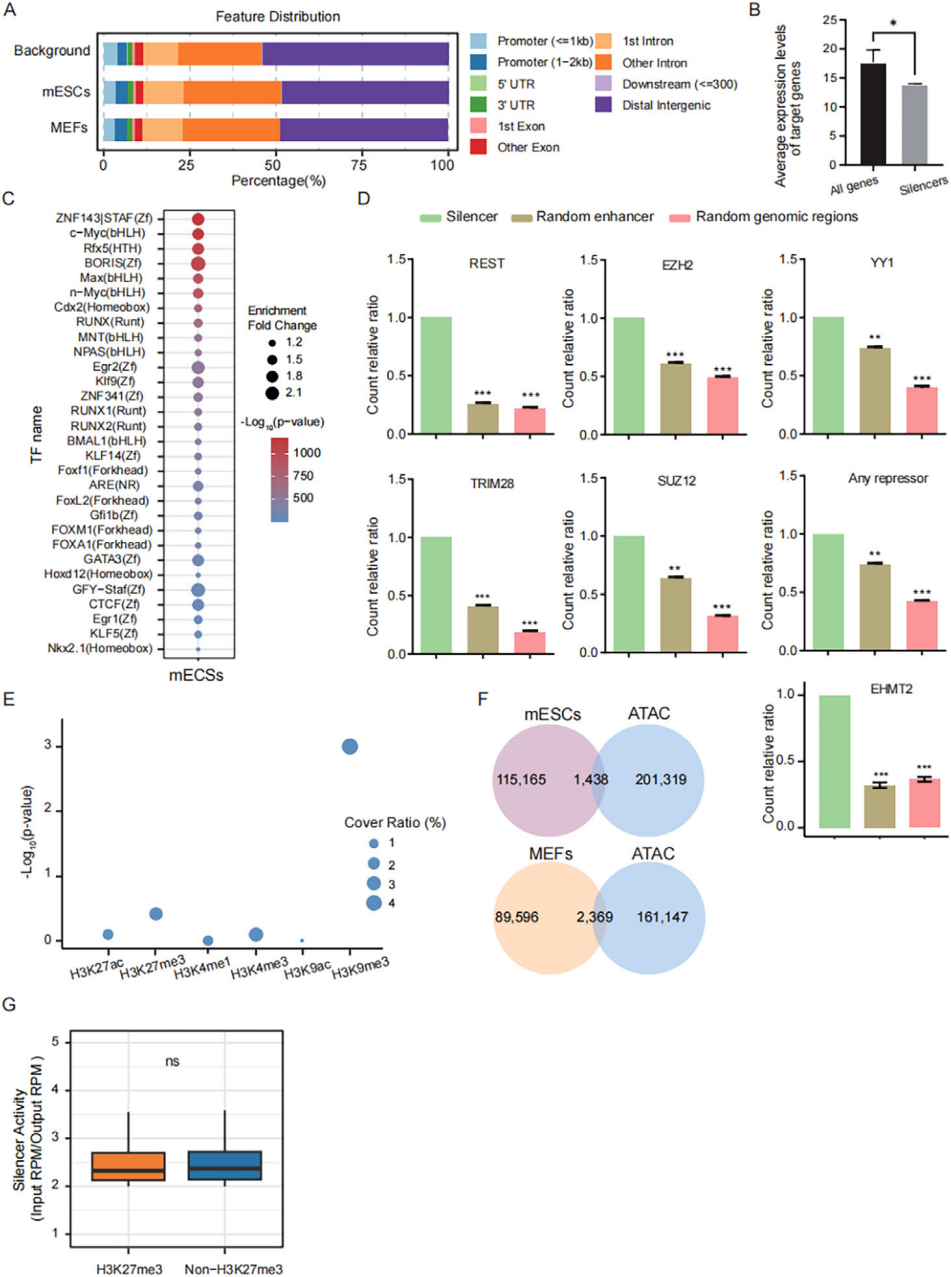
Distinct epigenetic features of mouse silencers. A) Genomic distribution statistics of silencers in mESCs, MEFs, and background. B) Comparison of the average expression levels between silencer‐associated target genes in mESCs and all genes across the genome. *p < 0.05 (calculated using *t*‐test). C) Bubble plots show TFs motif enrichment analysis of silencers in mESCs. D) Barplots presenting the count of silencers, random genomic regions, and random enhancers at known repressor TFBS based on ChIP‐seq data in mESCs. “Any repressor” refers to any one of REST, EZH2, YY1, TRIM28, or SUZ12(**p < 0.01, ***p < 0.001; data are presented as mean values ± SEM; *p* values are obtained using two‐sided Student's *t*‐test). E) Enrichment analysis of multiple histone modification sites of silencers in mESCs. The size of the circle represents the enrichment multiple; the enriched background regions are random genomic regions. F) Overlap analysis of silencers in mESCs and MEFs with the corresponding ATAC data. G) Comparison of the activity differences between silencers with H3K27me3 modification and those without H3K27me3 modification in mESCs. ns: not significant.

To investigate the transcriptional regulatory characteristics of silencers, we first examined their genomic distribution in the two cell types. Our analysis revealed that silencers predominantly reside in distal intergenic and intronic regions (**Figure** **2**A), mirroring the distribution patterns observed in human silencers.^[^ ^11^ ^]^ To investigate whether these silencers are associated with low gene expression, we adopted an analytical approach from existing literature,^[^ ^17^ ^]^ designating the nearest gene to each silencer as its target. The findings indicated that genes associated with silencers exhibited significantly lower expression levels compared to the genomic average (Figure 2B; Figure S2A, Supporting Information), highlighting a strong correlation between silencers and reduced gene expression. As enhancers are known to exert their *cis*‐regulatory functions by recruiting sequence‐specific transcription factors (TFs), we performed TF motif enrichment analyses to investigate whether specific TFs also play a crucial role in silencer activation. Our results demonstrated a notable enrichment of motifs linked to the zinc finger and Fox families, both known for their gene‐repressive functions(Figure 2C; Figure S2B, Supporting Information).^[^ ^3^, ^18^ ^]^ To further validate whether these silencers function in their native context, we conducted ChIP‐nexus experiments of ZNF143 in mESCs, and then, analyzed the overlap with silencers, enhancers, and random regions; the results showed a greater overlap of ZNF143 with silencers (Figure S2C, Supporting Information). For further verification, we silenced the expression of *Zfp143* using RNA interference (Figure S2D, Supporting Information) and performed a dual‐luciferase assay to measure the activity of ten silencers containing the ZNF143 motif. The results indicated that eight of these silencers exhibited a significant decrease in activity (Figure S2E, Supporting Information), suggesting that ZNF143 indeed enhances the repressive function of these silencers.

We also overlapped ChIP‐seq data of well‐known repressive TFs, such as REST, YY1, SUZ12, EZH2, and TRIM28, with silencers, enhancers, and random regions, respectively. This analysis revealed a higher frequency of these TFs at silencer sites, suggesting that silencers are preferentially bound by repressive TFs to modulate gene expression (Figure 2D). Enhancers are associated with distinct histone modifications such as H3K4me1 and H3K27ac.^[^ ^15^, ^19^ ^]^ To determine whether mouse silencers are marked by specific histone modifications, we integrated histone modification data for MEFs and mESCs from the modENCODE database with our identified silencers. Our findings revealed significant enrichment of H3K9me3, a repressive histone mark integral to gene regulation during development, in both cell types (Figure 2E; Figure S3A, Supporting Information).^[^ ^20^ ^]^ This suggests that H3K9me3 may serve as a hallmark of silencers in the mouse genome. We then analyzed the open chromatin region of silencers in the two cell lines, and the results showed that only ≈2% of silencers were in the open region (Figure 2F), which is consistent with the significant enrichment of H3K9me3 in silencers. Although previous studies have associated H3K27me3 with silencer activity,^[^ ^21^ ^]^ our data did not show a significant enrichment of H3K27me3 in mouse silencers (Figure 2E). Further investigation into the relationship between H3K27me3 and silencer function revealed no substantial increase in silencer activity with H3K27me3 modification compared to other silencers (Figure 2G). In summary, our findings offer comprehensive whole‐genome insights into the genomic and epigenomic characteristics of mouse silencers.

### 2.3 Cell‐Specificity of Silencers in the Mouse Genome

**Figure 3:**
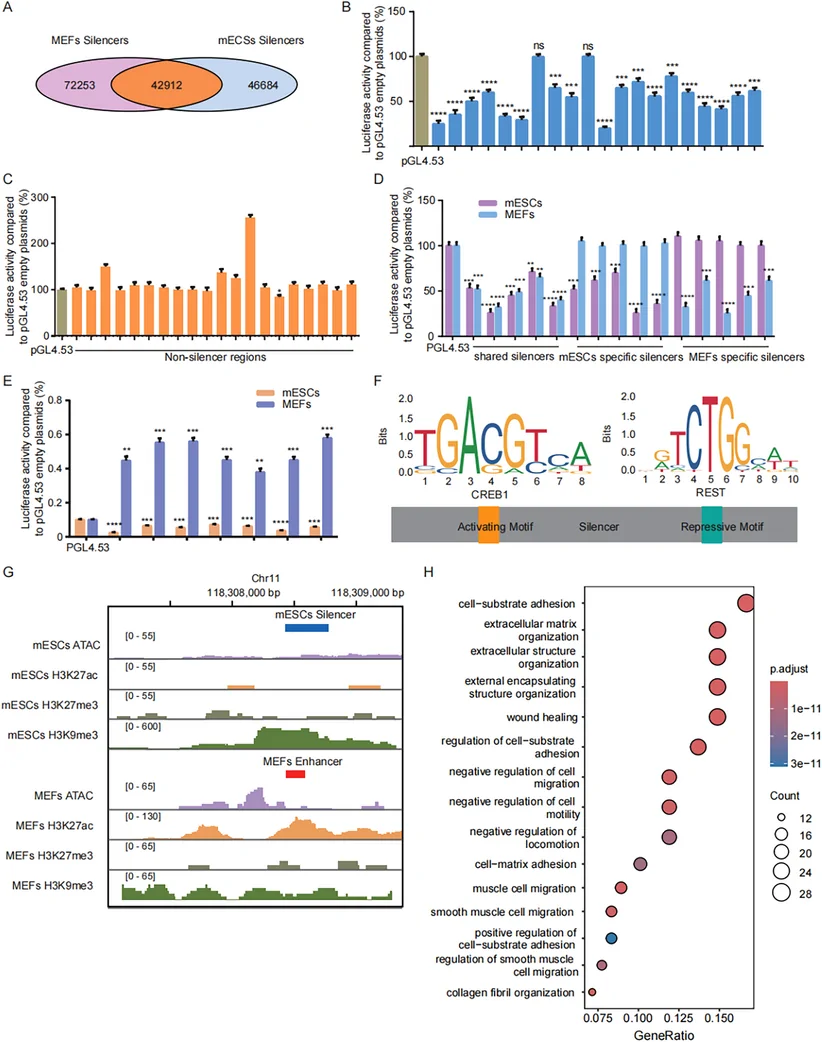
Cell‐type specificity of mouse silencers. A) Overlap analysis of silencers in mESCs and MEFs. B) False positive validation of silencers in mESCs. Twenty silencers were randomly selected based on the activity distribution for dual‐luciferase assays. Empty pGL4.53 plasmid was used as the control for baseline luciferase activity, and *y* axis represents the percentage of luciferase activity compared to pGL4.53 empty plasmids in the respective cells (***: *p* < 0.001, ****: *p* < 0.0001, ns: not significant; data are presented as mean values ± SEM; *p* values were obtained using two‐sided Student's *t*‐test, *n* = 3 biologically independent samples). C) False negative validation of silencers. Twenty sequences that were not identified as silencers in mESCs were randomly selected from the input library for dual‐luciferase assays in mESCs. Empty pGL4.53 plasmid was used as the control for baseline luciferase activity, and *y* axis represents the percentage of luciferase activity compared to pGL4.53 empty plasmids in the respective cells (*: *p* < 0.05, data are presented as mean values ± SEM; *p* values were obtained using two‐sided Student's *t*‐test, *n* = 3 biologically independent samples; only perform statistical analysis on the effect of silence). D) Verification of cell‐specific silencers. Five silencers of each type were selected and luciferase activity was detected in two cells. Empty pGL4.53 plasmid was used as the control for baseline luciferase activity, and *y* axis represents the percentage of luciferase activity compared to pGL4.53 empty plasmids in the respective cells (***: *p* < 0.001 and ****: *p* < 0.0001; data are presented as mean values ± SEM; *p* values were obtained using two‐sided Student's *t*‐test, *n* = 3 biologically independent samples). E) Six sequences identified as silencers in mESCs and reported as enhancers in MEFs were selected for activity validation in both cell types. Empty pGL4.53 plasmid was used as the control for baseline luciferase activity, and *y* axis represents the percentage of luciferase activity compared to pGL4.53 empty plasmids in the respective cells (**: *p* < 0.01, ***: *p* < 0.001, and ****: *p*<0.0001; data are presented as mean values ± SEM; *p* values were obtained using two‐sided Student's *t*‐test, *n* = 3 biologically independent samples). F) Presentation of activating and repressive motifs on bifunctional silencer sequences. G) Changes in ATAC, H3K27ac, H3K27me3, and H3K9me3 corresponding to the transition of bifunctional silencers to enhancers. H) The bubble chart illustrates the biological functional pathways associated with mESC‐specific silencers. The nearest gene of the silencer was used as the target gene.

To validate whether silencers exhibit cell specificity, we performed an overlap analysis of silencers in mESCs and MEFs. Over half of the silencers exhibited cell‐type‐specific presence in these cell types (**Figure** **3**A). To confirm the biological relevance of this specificity, we randomly selected 20 silencers regions (Table S1, Supporting Information) for luciferase assays. The results showed significant silencing activity in 18 out of the 20 silencers (Figure 3B). A strong correlation between Ss‐STARR‐seq activities and luciferase activity (Figure S3B, Supporting Information) corroborated the high stability of Ss‐STARR‐seq, as previously demonstrated (Figure 1D; Figure S1B, Supporting Information). We next assessed the false‐negative rate in our silencer identification. Twenty regions (Table S1, Supporting Information) not identified as silencers in the input library were randomly selected for dual‐luciferase assays. Only one region exhibited weak silencer activity (Figure 3C). To further confirm cell specificity, we tested both shared and cell‐specific silencers in both cell types. Shared silencers showed activity in both cell types, whereas cell‐specific silencers were active only in their respective cell types (Figure 3D). These findings collectively demonstrate that cell specificity is an intrinsic characteristic of mouse silencers.

Through a luciferase assay, we found that a silencer in one cell type exhibited enhancer activity in another cell type (Figure 3E). This indicates that CREs (*cis*‐regulatory elements) can undergo functional conversion depending on the cellular context. We discovered that these bifunctional silencers contained both repressive and activating motifs (Figure 3F), and their specific functionality (enhancer or silencer) was correlated with the expression levels of TFs (transcription factors) within the cells (Figure S3C, Supporting Information). We further investigated whether the conversion between enhancer and silencer was accompanied by changes in epigenetic features, and as expected, corresponding shifts in histone marks were observed (Figure 3G).

To explore the biological processes associated with the target genes of these silencers, we linked silencers to their nearest genes and conducted GO analysis. mESC‐specific silencers were enriched in pathways related to fibroblast‐associated processes, such as cell‐substrate adhesion and extracellular matrix organization, which are known to be repressed in mESCs (Figure 3H).^[^ ^22^ ^]^ In MEFs, specific silencer‐associated genes were primarily involved in transcription and translation‐related processes (Figure S2F, Supporting Information), which were expected to be suppressed in highly differentiated cells like MEFs.^[^ ^23^ ^]^ In addition, we performed a GO analysis on the silencer target genes shared between MEFs and mESCs. The results indicate that the enriched biological processes were not specific to either MEFs or mESCs (Figure S3D, Supporting Information).

### 2.4 Knockout of *Nanog* Silencer Significantly Enhances iPSCs Induction Efficiency

**Figure 4:**
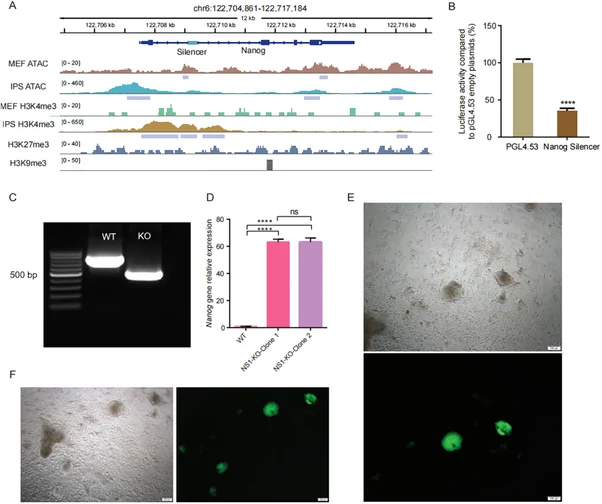
Knockout of *Nanog* silencers significantly increases the induction efficiency of iPSCs. A) Genomic location of *Nanog* silencers and corresponding ATAC, H3K27me3, and H3K9me3 signal profiles. B) Validate the activity of *Nanog* silencers using a dual‐luciferase assay. Empty pGL4.53 plasmid is used as the control for baseline luciferase activity, and *y* axis represents the percentage of luciferase activity compared to pGL4.53 empty plasmids in the respective cells (****: *p* < 0.0001; data are presented as mean values ± SEM; *p* values were obtained using two‐sided Student's *t*‐test, *n* = 3 biologically independent samples). C) Knockout(KO) silencers from gene *Nanog*. PCR result showing the removal of the *Nanog* silencer in two MEF cell clones, which is the representative result of experiments. The blots are cropped. D) Use qPCR to detect changes in *Nanog* gene expression in knockout cell lines (****: *p* < 0.0001; data are presented as mean values ± SEM; *p* values were obtained using two‐sided Student's *t*‐test, *n* = 3 biologically independent samples). E) NS1‐KO‐MEFs‐clone1 iPSCs and F) NS1‐KO‐MEFs‐clone 2 iPSCs and assess their OCT4 fluorescence expression. Use the iCD1 induction system to induce silencer knockout cell lines. On day 4 of induction, observe the colonies and EGFP‐OCT4 expression.

**Figure 5:**
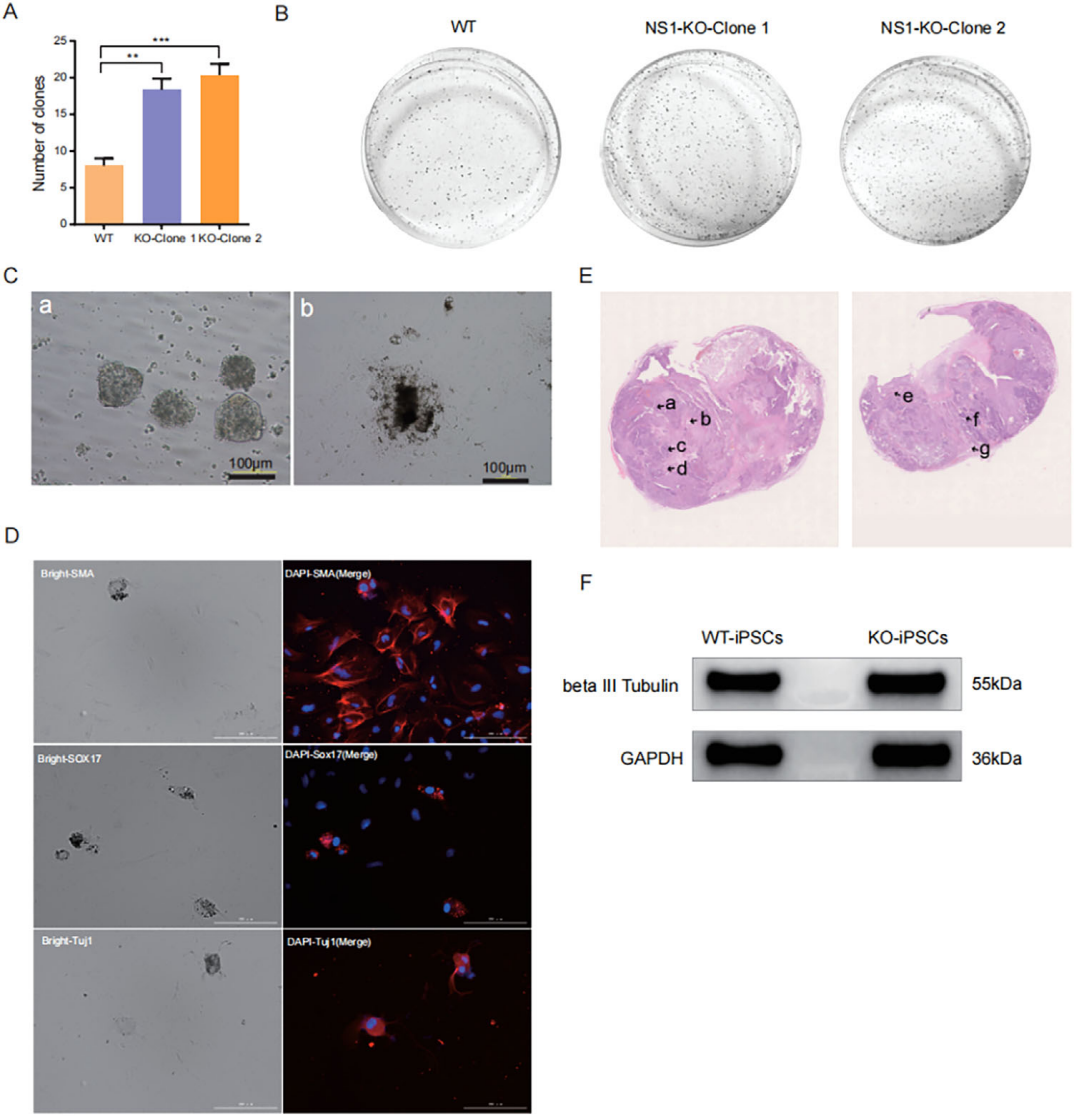
Assess the pluripotency of iPSCs induced from silencer knockout cell lines. A) Quantify the number of colonies in one field of view on day 7 of induction for both wild‐type and silencer knockout MEFs (**: *p* < 0.01, data are presented as mean values ± SEM; *p* values were obtained using two‐sided Student's *t*‐test, *n* = 3 biologically independent samples). B) On the 10th day of induction, alkaline phosphatase staining is used to visualize the colonies formed in wild‐type and silencer‐knockout two MEF clone replicates. C‐a) EBs formed by cloning‐like cells in suspension culture for 7 days. C‐b) Allow the EBs formed in condition (a) to continue adherent culture for spontaneous differentiation. D) Perform immunofluorescence staining for three germ layer marker proteins on spontaneously differentiated EBs. E) Conduct teratoma formation assays and H&E staining on iPSCs induced from silencer knockout cell lines (see details in the “Experimental Section”). E‐a) cartilage/mesoderm, E‐b) fat and surrounding fibrous tissue/mesoderm, E‐c) neural tube/ectoderm, E‐d) intestinal glandular epithelium/endoderm, E‐e) glandular epithelium/endoderm, E‐f) immature neural tube/ectoderm, and E‐g) fat and fibrous tissue/mesoderm. F) Western Blot results of beta III‐tubulin expression. WT‐iPSCs: iPSCs induced from wild‐type MEFs. KO‐iPSCs: iPSCs induced from NS1 silencer knockdown MEFs.

To further elucidate the role of silencers in key biological processes such as iPSCs induction, we investigated a putative silencer, NS1, located in the intronic region of the pluripotency gene *Nanog* and unrelated to H3K27me3 (**Figure** **4**A). Dual‐luciferase assays confirmed that NS1 exhibited significant repressive activity (Figure 4B). Using CRISPR technology, we knocked out NS1 in OG2‐MEFs, obtaining knockout clones (Figure 4C). qPCR analysis revealed a significant upregulation of *Nanog* expression in the knockout cells (Figure 4D), indicating that NS1 represses *Nanog* expression in MEFs. Simultaneously, we examined the top ten most likely off‐target sites, and the results showed no changes at these sites in the knockout cell line compared to wild‐type MEFs (Figure S4, Supporting Information). Given the essential role of *Nanog* in iPSC generation,^[^ ^24^ ^]^ we evaluated the impact of NS1 knockout on iPSC induction efficiency using the iCD1 system.^[^ ^25^ ^]^ Notably, while wild‐type MEFs formed colonies by Day 7, NS1 knockout cells began forming colonies as early as Day 4, with these colonies expressing E‐GFP fluorescence (Figure 4E, F). By Day 7, the number of colonies induced from knockout cells was significantly higher than that from wild‐type cells (**Figure** **5**A). The same result was obtained by alkaline phosphatase staining experiment (Figure 5B). These results suggest that knockout of the *Nanog* silencer substantially enhances reprogramming efficiency. To investigate the changes in key pluripotency genes during the reprogramming process, we measured the expression patterns of *Oct4*, *Sox2*, and *Nanog* in wild‐type and knockout cell lines on days 0, 4, and 7 upon induction. In the knockout cell line, expression levels of *Oct4*, *Sox2*, and *Nanog* were higher on Days 4 and 7 compared to the wild‐type, with the most pronounced difference observed on Day 4 (Figure S3E, Supporting Information). Among these genes, *Nanog* showed the greatest change in expression, likely due to its initially higher expression in the knockout cell line (Figure 4D).

To evaluate the in vitro differentiation potential of silencer‐knockout iPSCs, we cultured selected colonies in suspension, forming embryoid bodies (EBs) after 7 days (Figure 5C). Adherent culture of the EBs allowed for spontaneous differentiation, with immunofluorescence analysis revealing expression of the three germ layer marker proteins SOX17, SMA, and TUJ1 (Figure 5D). Further, teratoma formation assays demonstrated the presence of tissues corresponding to the three germ layers, confirming the in vitro differentiation capabilities of silencer‐knockout iPSCs (Figure 5E). Further, iPSCs generated from silencer‐knockout MEFs demonstrated no significant differences in embryoid body formation (Figure S5A, Supporting Information), trilineage differentiation potential (Figure S5B, Supporting Information), or teratoma formation capacity (Figure S5C, Supporting Information) when compared to iPSCs derived from wild‐type MEFs.

To investigate whether there are differences in the differentiation efficiency between wild‐type iPSCs and silencer‐knockout iPSCs, we directed their differentiation into neuronal cells, and subsequently, assessed the expression levels of beta III‐tubulin. The results showed no significant difference in beta III‐tubulin expression levels between the two groups (Figure 5F; Figure S3F, Supporting Information). In summary, knockout of the *Nanog* silencer significantly enhances *Nanog* expression and improves the efficiency of iPSCs induction, highlighting its crucial role in regulating pluripotency.

### 2.5 Knockout of *Smad2* Silencers Promotes Differentiation of Mouse Embryonic Stem Cells

**Figure 6:**
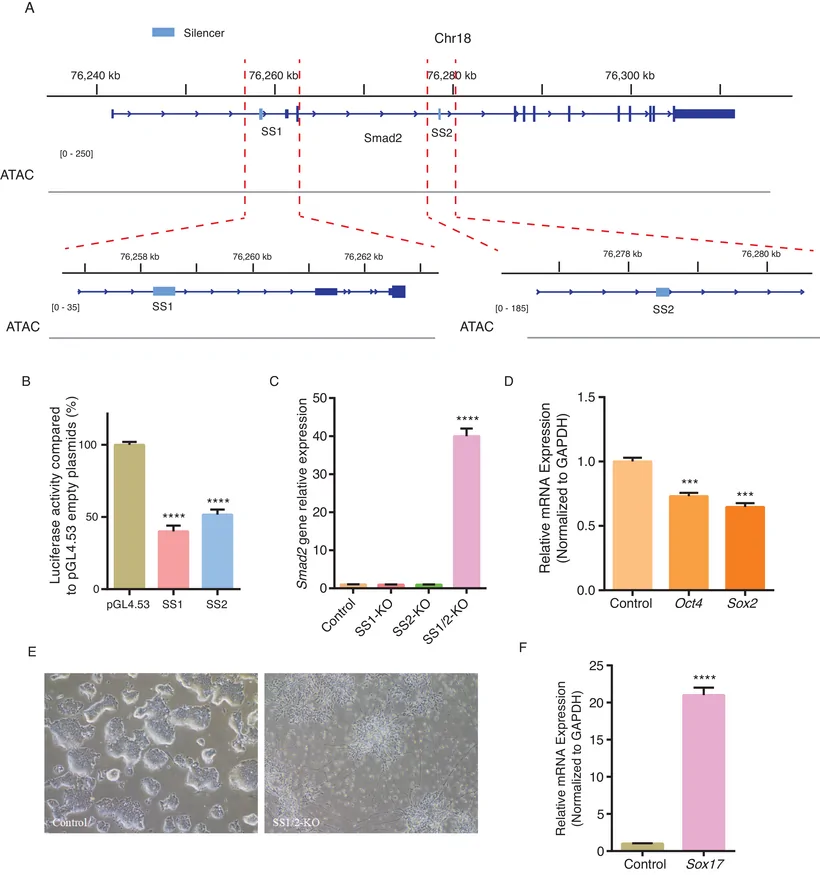
SS1 and SS2 silencers regulate *Smad2* gene expression and pluripotency in mESCs. A) Genomic location of *Smad2* silencers and corresponding ATAC signal profiles. B) Dual‐luciferase assay validation of SS1 and SS2. Empty pGL4.53 plasmid was used as the control for baseline luciferase activity, and *y* axis represents the percentage of luciferase activity compared to pGL4.53 empty plasmids in the respective cells (****: *p* < 0.0001, data are presented as mean values ± SEM; *p* values were obtained using two‐sided Student's *t*‐test, *n* = 3 biologically independent samples). C) qPCR analysis of *Smad2* expression changes in mESCs upon single knockout of SS1 or SS2 and double knockout of SS1 and SS2 (****: *p* < 0.0001; data are presented as mean values ± SEM; *p* values were obtained using two‐sided Student's *t*‐test, *n* = 3 biologically independent samples; control: mESCs subjected to CRISPR editing but without knockout of SS1 or SS2; all instances of “Control” in Figure 6 carry the same meaning). D) qPCR analysis of changes in the expression of pluripotency genes *Oct4* and *Sox2* in mESCs upon double knockout of SS1 and SS2 (***: *p* < 0.001; data are presented as mean values ± SEM; *p* values were obtained using two‐sided Student's *t*‐test, *n* = 3 biologically independent samples). E) Changes in mESC cell morphology upon double knockout of SS1 and SS2. F) qPCR analysis of changes in the expression of the endodermal gene *Sox17* in mESCs upon double knockout of SS1 and SS2 (****: *p* < 0.0001; data are presented as mean values ± SEM; *p* values were obtained using two‐sided Student's *t*‐test, *n* = 3 biologically independent samples).

To validate silencer‐mediated transcriptional regulation in mESCs, we identified two silencers located within the intronic region of the *Smad2* gene, both of which reside in open chromatin regions. We named them SS1 and SS2, respectively (**Figure** **6**A). We then assessed their silencer activity, and dual‐luciferase assays demonstrated that both SS1 and SS2 exhibit significant silencer activity (Figure 6B). To investigate whether SS1 and SS2 play a role in regulating gene expression, we performed knockouts of these elements in mESCs (Figure S5D, Supporting Information). The results showed that single knockout of SS1 or SS2 did not cause significant changes in Smad2 expression. However, when both SS1 and SS2 were knocked out simultaneously, Smad2 expression was significantly upregulated (Figure 6C), suggesting that SS1 and SS2 may have a synergistic effect in co‐regulating Smad2 expression. Similar phenomena were observed in enhancer^[^ ^18^ ^]^ and human silencer^[^ ^13^ ^]^ studies. Previous studies have reported that Smad2 influences the pluripotency of embryonic stem cells.^[^ ^26^ ^]^ Therefore, we examined the expression of pluripotency genes, and the results showed a significant downregulation of *Oct4* and *Sox2* expression (Figure 6D). In addition, when SS1 and SS2 were knocked out, mESCs failed to maintain normal colony morphology and began to differentiate (Figure 6E). Notably, we detected upregulation of the endodermal marker gene *Sox17* (Figure 6F), indicating that mESCs may be differentiating toward the endoderm. In conclusion, in mESCs, SS1 and SS2 co‐regulate *Smad2* expression and play a role in maintaining the pluripotency network.

## 3 Discussion

Although silencers were discovered ≈40 years ago, they remain under‐studied CREs.^[^ ^5^ ^]^ Recently, several high‐throughput methods for silencer identification have emerged. However, the limited overlap among silencers identified by different methods has impeded a comprehensive understanding of their characteristics. Similar to high‐throughput enhancer identification, silencer identification is challenged by the cell type‐specific activity of candidate sequences.^[^ ^3^ ^]^ To systematically summarize existing silencers, Zeng W. et al.^[^ ^27^ ^]^ developed the SilencerDB database, which includes 5 million silencers predicted by machine learning models and 33 000 silencers identified through high‐throughput or low‐throughput experimental screening. While such a compilation is valuable for silencer research, it is noteworthy that only 353 of these have been validated to exhibit silencing activity in vitro. Computational predictions based on epigenetic features and experimental methods for identifying regulatory elements have their limitations. For instance, even widely characterized enhancers often fail to exhibit activity in validation experiments.^[^ ^2^, ^28^ ^]^ Therefore, unbiased and systematic identification of silencers across the entire genome is crucial for elucidating their characteristics.^[^ ^3^ ^]^ In this study, we modified Ss‐STARR‐seq to enable unbiased, systematic quantification of silencer activity across the entire genome. Using this approach, we identified ≈200 000 silencer sequences in the mouse cell genome. Our collection of candidate fragments covered over 80% of the mouse genome with an average depth exceeding 11×. Notably, among the 20 non‐silencer sequences tested, only one exhibited silencer activity, indicating that we had screened the vast majority of silencers in the mouse genome. To date, no high‐throughput experimental methods have been reported that identify genome‐wide silencers in mammals.

The dual functionality of regulatory elements is a compelling area of study. In our investigation, we discovered that silencers in MEFs exhibit enhancer activity in mESCs. Notably, just 2 years after the term “silencer” was defined,^[^ ^5^ ^]^ it was reported that the same sequence could enhance transcription in vitro.^[^ ^29^ ^]^ Current research indicates that the dual functionality of CREs is relatively common;^[^ ^30^ ^]^ yet, this phenomenon remains underexplored.

Our findings reveal that these bifunctional silencer sequences contain both repressive and activating motifs, suggesting that the function of regulatory elements may depend on the TFs that bind to them in different cellular contexts. In addition, some observations provide insights into the bifunctionality of these silencers. For example, weak enhancer states are significantly enriched in human silencers,^[^ ^11^ ^]^ and some silencers also display active histone modifications such as H3K4me1 and H3K27ac.^[^ ^8^, ^10^, ^13^ ^]^ Zhu et al.^[^ ^18^ ^]^ noted that many enhancer functions were not correctly annotated due to resolution limitations, raising questions about whether enhancers and silencers are truly distinct entities. These bifunctional elements might comprise adjacent silencers and enhancers, with their function in different cell types dependent on the specific combination of TFs.^[^ ^3^, ^31^ ^]^

Histone modifications have long been associated with the function of regulatory elements. Enhancers, for example, are marked by active histone modifications such as H3K4me1 and H3K27ac.^[^ ^8^, ^10^, ^13^ ^]^ Conversely, H3K27me3 has been associated with silencers, and various studies have used H3K27me3 ChIP‐seq to identify these elements.^[^ ^9^, ^32^ ^]^ However, recent high‐throughput analyses suggest that H3K27me3 is not significantly enriched in the silencers of human cells; instead, H4K20me1 is more prevalent.^[^ ^11^ ^]^ Notably, H4K20me1 is linked to both transcriptional repression and activation; yet, its absence in *S. cerevisiae* indicates that it is not a universal marker for silencers across eukaryotes. Our study found that silencers in two mouse cell lines were significantly enriched in H3K9me3, reinforcing this observation. Our results also revealed that silencers are not enriched for H3K27me3 but significantly overlap with the core subunits of PRC2, EZH2 and SUZ12. To further investigate, we performed an overlap analysis between H3K27me3‐mediated silencers and PRC2‐mediated silencers, finding that their overlap was ≈5% (Figure S3G, Supporting Information). This result suggests that H3K27me3‐mediated silencers and PRC2‐mediated silencers are functionally independent.

Leveraging the episomal‐based silencing profiling capability of Ss‐STARR‐seq, we systematically categorized silencers into two distinct classes: 1) those embedded within heterochromatin domains and 2) those located in open chromatin regions. While silencers in open chromatin regions have been documented, their functional validation remains limited.^[^ ^3^, ^33^ ^]^ Here, we validated the transcriptional repressing role of such silencers, including NS1, SS1, and SS2—all lacking H3K9me3 modification; yet, situated within open chromatin landscapes—highlighting the endogenous regulatory potential of such type of silencers. Further, luciferase reporter assays showed that silencers with H3K9me3 modifications generally exhibited weaker repressive activity (Figure S5E, Supporting Information), whereas those located in open chromatin regions displayed stronger repressive activity (Figure S5F, Supporting Information). As Ss‐STARR‐seq assesses the silencing potential of sequences, we propose that silencers within heterochromatin possess latent episomal silencing potential, whereas those in open chromatin exhibit robust regulatory activity, likely driven by the exposure of sequences containing DNA‐binding motifs of repressive TFs.

Historically, the limited identification of silencers in each cell type, particularly the lack of genome‐wide data, has restricted the statistical analysis of their associated chromatin features. Further, the pronounced cell specificity and functional variability of silencers complicate their characterization. It is plausible that silencers are not defined by a single histone mark but rather by a combinatorial code of multiple histone modifications unique to each cell type.

Studying the biological effects of CREs is a key objective in functional genomics. For example, experimental evidence underscores the pivotal roles of enhancers in the growth and development of organisms.^[^ ^34^ ^]^ Similarly, the biological impacts of silencers have been explored, with Pang et al. demonstrating that silencer knockout increased drug resistance in K562 cells.^[^ ^11^ ^]^ However, there has been a lack of studies examining the role of individual CREs in complex biological processes, such as the induction and maintenance of embryonic pluripotency. In our study, we significantly enhanced the induction efficiency of iPSCs by knocking out the silencer of the pluripotency gene *Nanog*, and experimentally confirmed the full pluripotency of these iPSCs. Although the NS1 silencer sequence is absent from the genome of these iPSCs, the *Nanog* gene remains repressed during differentiation. This phenomenon may resemble the plasticity observed in enhancer functionality.^[^ ^35^ ^]^ A potential mechanism underlying silencer activity could involve dynamic interactions between a gene and silencers, which are not fixed but adapt to changes in cell type or environmental context. In this scenario, the gene may engage with alternative silencers to form new regulatory interactions, thereby modulating its expression.

As no silencers were identified in the intronic regions of *Oct4*, *Klf4*, and *Sox2* genes, we did not extend our detailed analysis to these genes. Literature suggests that silencers can modulate target gene transcription via long‐range interactions.^[^ ^11^ ^]^ Consequently, in future research, we plan to employ high‐resolution Hi‐C in MEFs to identify silencers interacting with *Oct4*, *Klf4*, and *Sox2*. The co‐knockout of potential silencer of these genes might further increase the efficiency of iPSCs induction.

## 4 Experimental Section

### Mouse Genome Input Library and Output Library Construction

The Ss‐STARR‐seq vector ligated with the genomic fragments was amplified through plasmid replication to construct the input plasmid library. This library was subjected to high‐throughput sequencing to ensure adequate genomic coverage. The validated input library was then transfected into mESCs and MEFs to construct the output library. The detailed procedures for input and output library preparation are referenced in the authors’ previously published preprint.^[^ ^25^ ^]^

### Cell Culture and Transfection

MEFs were inoculated with 10% FBS (GIBCO; Cat 10437028) and 1% penicillin streptomycin (P/S) (Sigma; cat P0781) in DMEM (Thermo Fisher; Cat 10566024); MEFs were induced in iCD1 medium (Delicell; Cat 820250). iPSCs and mESCs were cultured in N2B27‐2i/LIF medium (Neural Basal, F12, 1% Glutamax, 1% NEAA, 1% Sodium pyruvate, 0.1 mm β‐mercaptoethanol, 1% B27, 0.5% N2, 0.5% P/S [optional], mLif [1:8000], 3 um CHIR99021 [1:4000], and 1 um PD0325901). The culture conditions of all cells were 37 °C, 5% carbon dioxide. When the cell growth confluence was ≈80%, nucleofection electroporator (Lonza; Cat aAF‐1002X) was transfected into the input library (1 µg DNA per 10^6^ cells). mESCs were transfected using Lipofectamine 3000 Transfection Reagent (Invitrogen; Cat L3000075). The cells after 24 h of transfection were collected to construct the output library.

### Library Quality Control

The input and output libraries from Illumina sequencing underwent quality control. TrimGalore software was used for offline data quality control, which included adapter removal and the elimination of low‐quality bases and short sequences (parameters: ‐q20‐length 25). The cleaned data from this process were then aligned to the mouse reference genome mm10 using Bowtie2^[^ ^36^ ^]^ software to generate a BAM file. Sambamba software^[^ ^37^ ^]^ was employed to remove PCR duplicate reads, followed by the use of SAMtools^[^ ^38^ ^]^ software to extract the BAM file containing properly paired reads.

### Identification of Mouse Silencers

To minimize the impact of fragment loss during the transfection and sequencing processes, statistical tests were first conducted only on bins that contained more than 20 reads. Clean data were aligned to mm10 genome using bwa, and a BAM file was generated through SAMtools. Using the BAM file of the input and output libraries, which included only well‐aligned reads at both ends, conversion into a bw file (normalized using None) was done via the bamCoverage command of Deeptools. Next, the CRADLE software was used to identify silencer regions across the whole genome. The first step involved employing the correctBias_stored function to correct for technical biases in read counts (such as shearing, PCR, mappability, and G‐quadruplex). Following this, the callPeak command was used to identify DNA regions with regulatory activity and inhibitory effects throughout the genome (for more details, see CRADLE on PyPI). The identified regions with inhibitory effects were designated as silencer regions. The specific parameter settings used were as follows: ‐rbin 300, ‐wbin 100, ‐d 20, and ‐fdr 0.05. The key parameters were explained as follows: ‐rbin: the bin size used for defining regions, which cannot be smaller than wbin; ‐wbin: the bin size used for testing differential activity, which cannot be larger than rbin; ‐d: the minimum distance between peaks (peaks closer than this value (in bp) are merged);and ‐fdr: FDR control. Silencer activity was measured by fold change, with fold change calculated as the ratio of Input RPM to Output RPM.

While obtaining the silencer results, ChipSeeker software^[^ ^39^ ^]^ was employed to create a genome‐wide distribution map of silencers and to enrich for multiple histone modification sites. Genes adjacent to silencers were identified as target genes and subjected to functional enrichment analysis using the R package clusterProfiler.^[^ ^40^ ^]^ Methylation signal and motif enrichment analysis were performed using Deeptools^[^ ^41^ ^]^ and HOMER^[^ ^42^ ^]^ software, respectively. Bar, pie, and Venn charts were generated using R version 4.2.1 based on the calculated data.

### Determination of the Final Number of Silencers

A custom script was used to extract the same number of background sequences as the silencer, and the background sequence length and GC content were consistent with the silencer. Deeptool software was used to count the number of reads in the sequence region and calculate the RPM value. Then, the fold change value of the silencer and background sequence were calculated according to the formula Input RPM/output RPM and normalized using log_2_. Using this method, a twofold or higher Ss‐STARR‐seq enrichment over input was observed in less than 3% of the randomly selected regions, and this was used as the cutoff for significant silencer signals. Statistics were plotted using R.

### Silencer‐Associated Gene Identification

Using BedTools window (v2.28.0), RefSeq genes within a ± 100 kb window around all silencer candidates were extracted.

### Library Repeatability Evaluation

To assess the reproducibility between different libraries, two approaches were employed. The first approach evaluated the consistency of read distribution across the entire genome; while, the second approach assessed the consistency of whole‐genome silencer activity. Both methods were implemented with Deeptools software. For the evaluation of genome‐wide read distribution consistency, the multiBamSummary bins command was used to divide the genome into 10 kb bins, count the number of reads in each bin, and then, correct for library depth. The plotCorrelation command was used to visualize the correlation between libraries. For evaluating the consistency of genome‐wide silencer activity across different libraries, the multiBamSummary BED‐file command was utilized to count the reads in silencer regions in different libraries. After correcting for library sequencing depth, the Input RPM/output RPM value was used to represent the activity intensity of each silencer. Using the activity intensity data for all silencers across different libraries, correlation analysis was performed and the silencer activity correlation between libraries was visualized using R packages ggplot2 and ggpubr.

### Genome Coverage Analysis of the Library

The coverage command from BEDtools^[^ ^43^ ^]^ was used to calculate the read coverage for each window (1 Mb) in the genome within the input library. Figures were generated using R (V4.2.1) based on the calculated data.

### iCD1 Induction System

Plat‐E cells were prepared a day before the experiment. They were digested with 0.25% trypsin and the cells counted. Plate Around 8 × 10^6^ cells were plated on a 100 mm dish. When the cells reached a 80–90% confluence, they were transfected with lipo3000 (Invitrogen; L3000075). After 12 h, the medium was replaced with 10 mL of DMEM containing 10% FBS. After another 24 h, MEFs were plated at a density of 5 × 10^4^ cells per well. The supernatant was collected from the Plat‐E cells after 12 h and filtered through a 0.45 µm membrane into a centrifuge tube. Polybrene was added to a final concentration of 8 µg mL^−1^, and the medium of MEFs was replaced with fresh medium. 1 mL of viral supernatant was added to each well (reduce proportionally if using concentrated virus), using Pmx‐mCherry virus as the control group. A second viral transfection was performed after 24 h, and after another 24 h, the medium was replaced with iCD1 mouse induction medium.

### q‐PCR Experiment

The cells were digested with 0.25% trypsin (GIBCO; Cat R00950) and washed twice with PBS to collect them. The total RNA of the cells was extracted with the kit (Vazyme, RC102). Each reaction was reversed with 1 µg total RNA and SYBR Green SuperMix (Vazyme, R323) on the 7500 Fast Real‐Time PCR System (Applied Bio‐systems). Using the reverse‐transcribed cDNA as a template, the appropriate primers (0.25 µm) were added and enzyme mixed, followed by gene expression analysis on the machine.

### Dual‐Luciferase Reporter Assay

The silencer sequences to be validated were synthesized by GENEWIZ, China. These sequences were inserted into the hPGK promoter of the luciferase plasmid pGL4.53 (Promega) via Gibson assembly. The reporter gene vector and the pGL4.53 vector with the inserted silencer sequences were then co‐transfected into the cells. Luciferase assays were performed according to the manufacturer's protocol using the Dual‐Glo Luciferase Assay System (Promega). The original luciferase plasmid without the inserted sequences served as the control. All luciferase assays were conducted in triplicate on different days using independent transfections. All luciferase bar graphs were generated using GraphPad software, and significance was analyzed using *t*‐tests.

### Silencer Knockout Experiment

First, knockout gRNAs targeting the 5′ and 3′ ends of the silencer were designed using the website http://crispor.tefor.net/. Then, the synthesized gRNAs were cloned into the PX459 V2 plasmid (Addgene) separately. The plasmids were co‐transfected into the cells. After cloning single‐cell colonies, cellular DNA was extracted, and PCR primers were designed based on the silencer location (Table S1, Supporting Information) for amplification and Sanger sequencing.

### In Vitro Differentiation Experiment

The culture of mouse iPSCs cells was suspended in N2B27‐2i/LIF medium for 7 days, followed by adherent culture to promote differentiation into cells of different germ layers.

### ChIP‐Nexus

ChIP‐nexus experiments were performed as previously described^[^ ^44^ ^]^ with slight modification. 10^7^ cells were crosslinked with 1% formaldehyde for 10 min at RT, and the reaction was quenched with 125 mm glycine. Crosslinked cells were lyzed using ChIP buffer (10mm Tris‐HCl pH 7.5, 1 mm EDTA, 1% Triton X‐100, 0.1% sodium deoxycholate, 150 mm NaCl, 1× protease inhibitors) and sonicated to fragment the DNA from 100 to 10 000 bp. Through centrifugation at 4 °C for 10 min, the supernatant was incubated with anti‐ZNF143 antibody overnight at 4 °C with slow rotation. Targeted chromatin was enriched with magnetic protein A/G beads. The beads were washed with washing buffer to remove the nonspecific binding. The beads‐DNA ends were blunted using NEB Next End Repair Module (NEB; Cat E6050S), and tail‐A was added with dATP using Klenow exo‐ (NEB; Cat M0212S). The beads‐DNA was ligated with the Nex adapter, and the 5′ overhang of the adaptor‐ligated DNA was filled using Phi29 Polymerase (Vazyme; Cat N106‐01). The blunt‐end DNA was treated with Lambda Exonuclease (NEB; Cat M0262S) to digest non‐protein‐binding DNA. After reverse‐crosslinking at 65 °C overnight, DNA was purified using phenol/chloroform and ethanol. The purified DNA was denatured to generate ssDNA at 95 °C. The ssDNA was self circularized with ssDNA Ligase (Epicenter; Cat CL4111K). The circular ssDNA was used for construction of the ChIP‐nexus library with illumina primers. The right DNA library was purified using gel‐purification and sequenced on an Illumina platform.

### ATAC‐Seq

All reagents used in this experiment were sourced from Novoprotein's reagent kit (Novoprotein; N248). To culture mouse iPSCs, 200 000 cells per replicate were harvested, and 50 µL of lysis buffer was added. The mixture was incubated on ice for 5 min, followed by the addition of 950 µL of wash buffer to obtain a nuclear suspension. 10 µL of the nuclear suspension was mixed with 10 µL of trypan blue dye, gently pipetted to mix, and 10 µL of the mixture was loaded onto a cell counting chamber for microscopic counting. Based on the nuclear count, 100 000 nuclei were then mixed with 40 µL of fragmentation buffer and incubated at 37 °C for 30 min. After incubation, 10 µL of STOP buffer was added, and the reaction was incubated at 50 °C for 5 min to terminate the reaction. Tagment DNA Extract Beads were used to purify the fragmented product. Following the kit instructions, the purified product underwent PCR amplification. To purify the PCR product, two volumes of Novoprotein DNA Clean Beads were added to the PCR product. Finally, 10 µL of the sample was taken for Illumina high‐throughput sequencing.

### Data Analysis of ChIP‐Nexus

Raw reads were trimmed using Cutadapt (v2.10)^[^ ^45^ ^]^ to remove the first 10 bp random barcode and adaptor sequences. The reads were aligned to the human reference genome GRCh38/hg38 using Bowtie2 (v2.3.5.1)^[^ ^36^ ^]^ and transformed into SAM file using Samtools (v1.12).^[^ ^38^ ^]^ Narrow peaks were called using MACS2 (v2.2.7.1).^[^ ^25^ ^]^

### Immunofluorescent Staining

The cell culture medium was removed and the cells were washed three times with PBS buffer. The cells were fixed with 4% paraformaldehyde for 30 min at room temperature. After fixation, the cells were washed three times with blocking solution (PBS containing 100 mmol L^−1^ glycine and 0.3% BSA). The cells were permeabilized by adding 1% Triton X‐100 solution and incubating at room temperature for 15 min. Then, they were blocked with 5% bovine serum albumin (BSA) for 2 h at room temperature. The cells were washed three times with TBP (Triton X‐100, BSA, PBS). The primary antibody was added and then overnight incubation was done at 4 °C. The next day, the cells were allowed to return to room temperature for 20 min; then, washed three times with TBP. The secondary antibody was added, followed by incubation for 1 h at room temperature. Finally, the cells were washed three times with TBP and observed under a fluorescence microscope.

### Teratoma Experiment

For the teratoma experiment, Cyagen C‐NKG severe immunodeficient mice (Cyagen) were used, with five female mice, aged 5–6 weeks. mIPS cells were digested to obtain a single‐cell suspension, washed twice with PBS buffer, and resuspended in PBS to achieve a final concentration from 5 × 10^7^ to 1 × 10^8^ mL^−1^. Each group was injected with 500 µL of the cell suspension. The mice were restrained and the cell suspension mixed well; slowly, 100 µL of the cell suspension was injected subcutaneously into the right anterior shoulder using a 1 mL syringe. The needle was withdrawn slowly to minimize cell leakage. The treated mice were labeled and their information was recorded. After 3 weeks, the subcutaneous tumor volumes were observed and recorded; then, the mice were euthanized by cervical dislocation. The teratomas were excised using scissors and forceps. The teratomas were immersed in 4% PFA for paraffin embedding and H&E staining. The stained sections were analyzed to assess the differentiation of the three germ layers.

## Conflict of Interest

The authors declare no conflict of interest.

## Supporting information



Supporting Information

Supporting Table 1

## Data Availability

The data that support the findings of this study are available from the corresponding author upon reasonable request.
