# Supplementary material for: Genome‐Wide Silencer Screening Reveals Key Silencer Modulating Reprogramming Efficiency in Mouse Induced Pluripotent Stem Cells
Source: Adv Sci (Weinh). 2025 Mar 20;12(18):2408839. doi: 10.1002/advs.202408839 (PMC12079485; doi:10.1002/advs.202408839)
Supplement: Supplementary file 1 — Supporting Information [file ADVS-12-2408839-s001.docx]

**Supplemental Information**

**Genome-wide Silencer Screening Reveals Key Silencer Modulating Reprogramming Efficiency in Mouse Induced Pluripotent Stem Cells**

Xiusheng Zhu^1^†, Lei Huang^1^†, Guoli Li^1^†, Biao Deng^1,2,3^†, Xiaoxiao Wang^1^†, Hu Yang^1^†, Yuanyuan Zhang^1^, Qiuhan Wen^1^, Chao Wang^1^, Jingshu Zhang^1^, Yuxiang Zhao^2^*, Kui Li^1^*, Yuwen Liu^1^*

This file includes:

Supplementary figure legends for figures S1-S5;


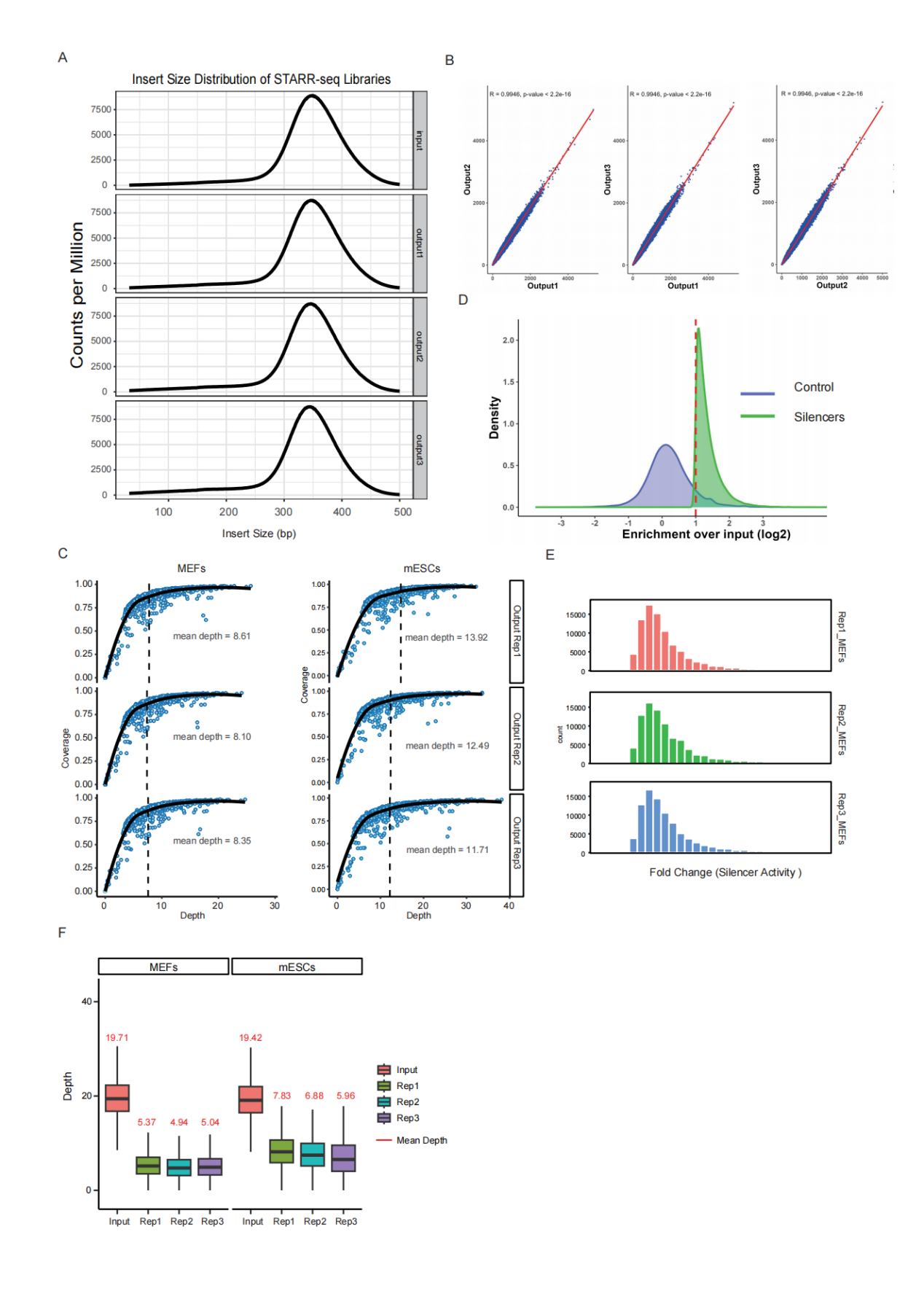


**Figure S1. Perform input and output-related assays for the identification of mouse silencers**.

1. Quantify the fragment sizes of input and output samples.
2. The correlation between the biological repeats of the MEFs output biological library was analyzed. The x, y coordinate axes represent the number of reads of the genomic bin in different libraries, and the color level indicates the degree of enrichment of the points, output 1/2/3/4 represents three output libraries with different repeats.
3. The coverage and depth of output library sequences in mESCs and MEFs.
4. Ss-STARR-seq enrichment at MACS2 peaks and randomly selected GC% and length matched regions. A 2-fold or higher STARR-seq enrichment over input (red dashed line) was observed in less than 3% of the randomly selected regions and used as a cutoff for significant silencer signal (MEFs). Regions with the same quantity, length and GC content of peaks were randomly selected in the genome. fold-change was calculated, and plotted using ggplot2 with R (V4.2.2).
5. The range of silencer activity (fold change) identified in output libraries across different replicates in MEFs.
6. The depth of input and output libraries for each replicate in mESCs and MEFs is displayed.


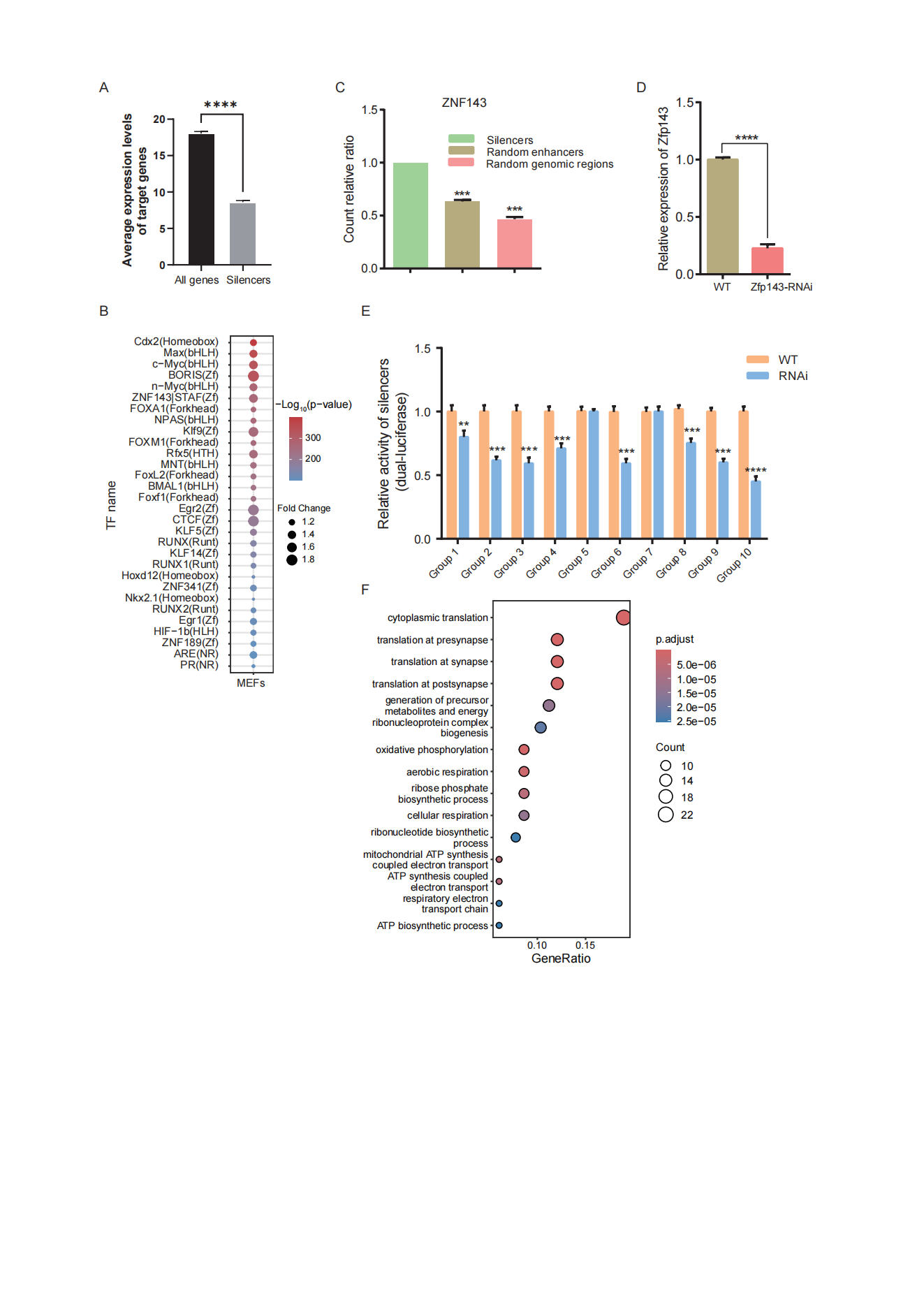


**Figure S2. Characteristics of silencers in MEFs and validation of ZNF143 binding to silencers in mESCs.**

1. Comparison of the average expression levels between silencer-associated target genes in MEFs and all genes across the genome. *****P* < 0.0001 (calculated using t-test).
2. Bubble plots showed TFs motif e*nrichm*ent analysis of silencers in MEFs.
3. Barplots presenting the count of silencers, random genomic regions and random enhancers at ZNF143 TF based on ChIP-seq data in mESCs (***: p<0.001, data are presented as mean values +/- SEM; P values were obtained using two-sided Student’s t-test).
4. The change in relative expression of the *Zfp143* gene in mESCs after interference, as detected by qPCR(****: p<0.0001, data are presented as mean values +/- SEM; P values were obtained using two-sided Student’s t-test).
5. The change in activity of 10 silencers containing the ZNF143 motif in *Zfp143*-interfered mESCs, as measured by dual-luciferase assay (**: p<0.01, ***: p<0.001, data are presented as mean values +/- SEM; P values were obtained using two-sided Student’s t-test).
6. The bubble chart illustrates the biological functional pathways associated with MEF-specific silencers. The nearest gene of the silencer was used as the target gene.


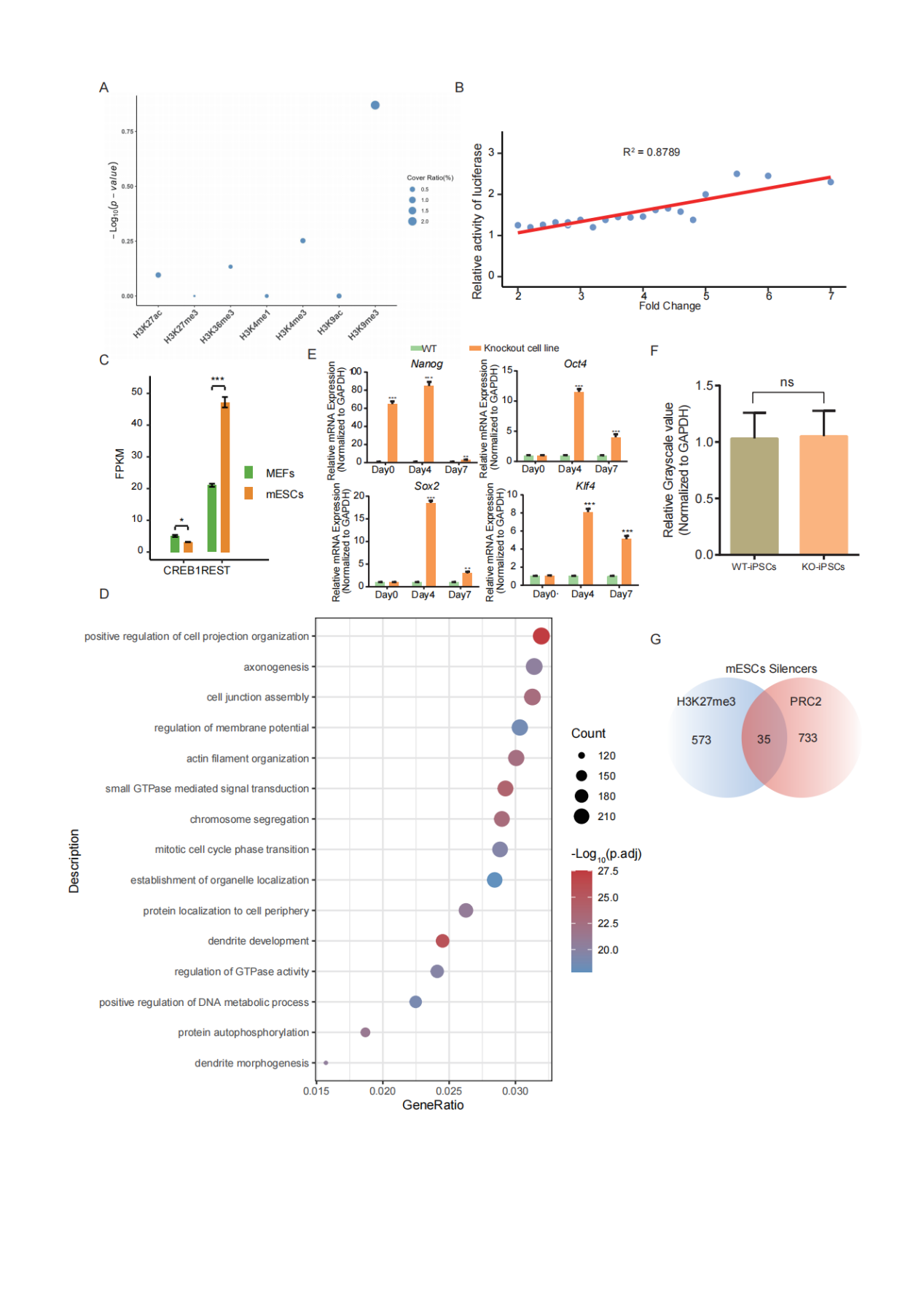


**Figure S3. Characteristics of histone modifications in MEF silencers and the results of alkaline phosphatase staining.**

1. Enrichment analysis of multiple histone modification sites of silencers in MEFs. The size of the circle represents the enrichment multiple, the enriched background regions are random genomic regions.
2. According to the results of Fig. 3B, the correlation between Ss-STARR-seq activity and luciferase activity was analyzed.
3. Comparison of expression levels of *Creb1* and *Rest* in mESCs and MEFs. * denotes P-value < 0.05, and *** denotes P-value < 0.001.
4. The bubble chart shows the biological process pathways associated with shared silencers in mESCs and MEFs.The nearest gene of the silencer was used as the target gene.
5. Compare the changes in pluripotency genes *Nanog*, *Oct4*, *Sox2* and *Klf4* at different induction time points between wild-type and silencer knockout MEFs (**: p<0.01, ***: p<0.001, data are presented as mean values +/- SEM; P values were obtained using two-sided Student’s t-test, n=3 biologically independent samples).
6. Quantification of the Western Blot band intensity in Figure 5C. ns: no significant difference.
7. The overlap between H3K27me3-mediated silencers and PRC2-mediated silencers in mESCs.


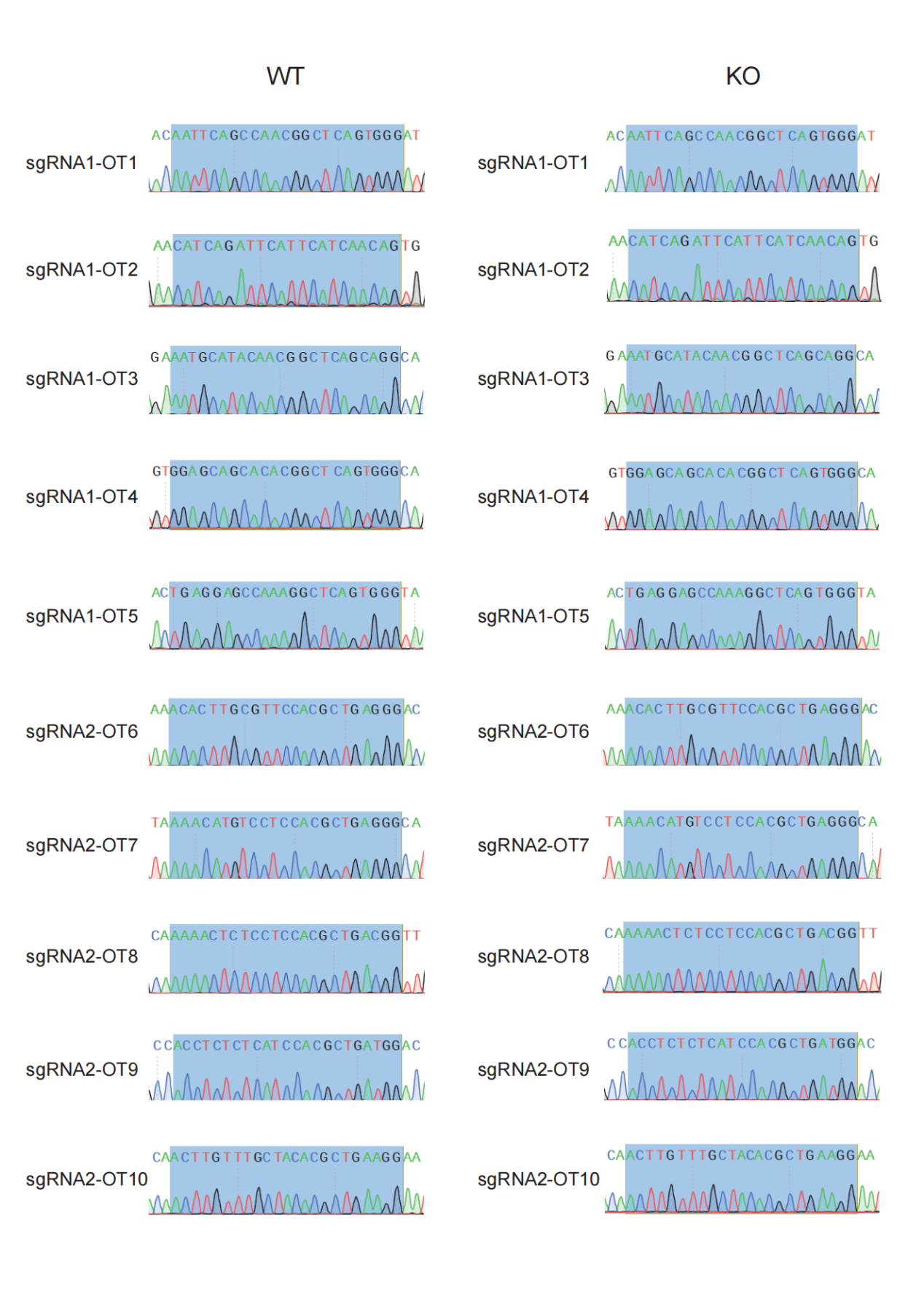


**Figure S4. Off-target effect detection of the knockout experiment.**

The image shows the Sanger sequencing results of the top 10 potential off-target sites in wild-type MEFs(WT) and silencer-knockout MEFs(KO).


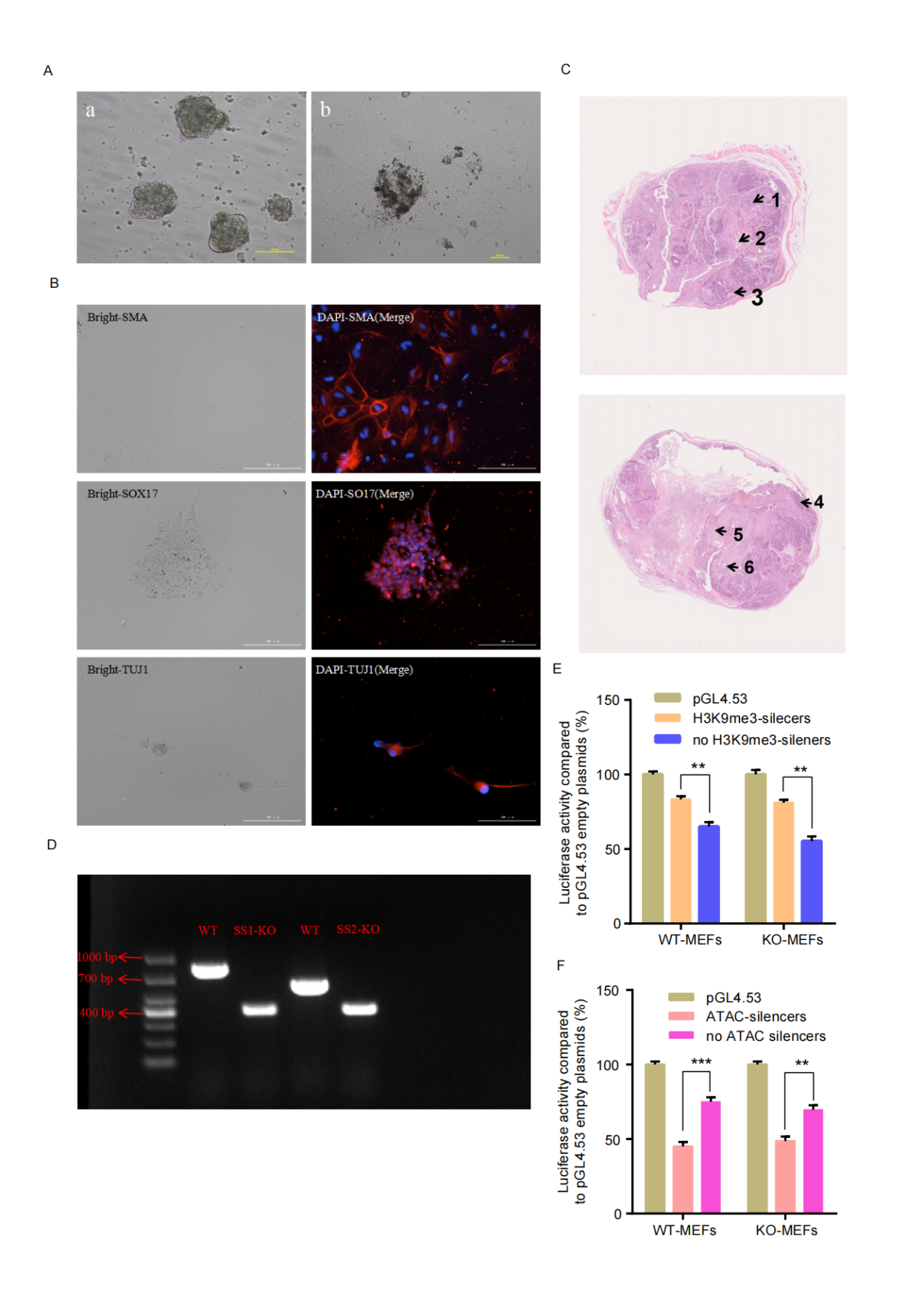


**Figure S5. Wild-type iPSCs differentiation capability assay.**

1. a). EBs formed by cloning-like cells in suspension culture for 7 days. b). Allow the EBs formed in condition (a) to continue adherent culture for spontaneous differentiation. B) Perform immunofluorescence staining for three germ layer marker proteins on spontaneously differentiated EBs. C) Conduct teratoma formation assays and H&E staining on wild-type iPSCs (see details in the "Experimental Section"). 1:Neural tube/ectoderm; 2:Vessels and surrounding fibrous/mesoderm; 3:Intestinal gland epithelium/ectoderm. 4:Neural tube/ectoderm; 5:Glandular epithelium/endoderm; 6: Cartilage components/mesoderm. D) PCR result showing the removal of the silencer in two mES cell clones, which is the representative result of two experiments. The blots were cropped. Full scans of the blots are shown in Table S2. E) The luciferase activity of silencers with or without H3K9me3 modification in KO-MEFs and WT-MEFs. Empty pGL4.53 plasmid was used as the control for baseline luciferase activity, and *y* axis represents the percentage of luciferase activity compared to pGL4.53 empty plasmids in the respective cells (**: *p*<0.01, data are presented as mean values +/- SEM; *P* values were obtained using two-sided Student’s *t*-test, n=3 biologically independent samples). F) The luciferase activity of silencers with or without ATAC modification in KO-MEFs and WT-MEFs. Empty pGL4.53 plasmid was used as the control for baseline luciferase activity, and *y* axis represents the percentage of luciferase activity compared to pGL4.53 empty plasmids in the respective cells (**: *p*<0.01, **:*p*<0.001, data are presented as mean values +/- SEM; *P* values were obtained using two-sided Student’s *t*-test, n=3 biologically independent samples).
